# Experimental evidence of pollination in marine flowers by invertebrate fauna

**DOI:** 10.1038/ncomms12980

**Published:** 2016-09-29

**Authors:** Brigitta I. van Tussenbroek, Nora Villamil, Judith Márquez-Guzmán, Ricardo Wong, L. Verónica Monroy-Velázquez, Vivianne Solis-Weiss

**Affiliations:** 1 Unidad Académica de Sistemas Arrecifales-Puerto Morelos, Instituto de Ciencias del Mar y Limnología, Universidad Nacional Autónoma de México, Prolongación Niños Héroes S/N, Puerto Morelos, Quintana Roo 77580, Mexico; 2 Laboratorio del Desarrollo en Plantas, Facultad de Ciencias, Universidad Nacional Autónoma de México, Circuito Exterior, Ciudad Universitaria, Del. Coyoacán, Ciudad de México 04510, Mexico

## Abstract

Pollen transport by water-flow (hydrophily) is a typical, and almost exclusive, adaptation of plants to life in the marine environment. It is thought that, unlike terrestrial environments, animals are not involved in pollination in the sea. The male flowers of the tropical marine angiosperm *Thalassia testudinum* open-up and release pollen in mucilage at night when invertebrate fauna is active. Here we present experimental evidence that, in the absence of water-flow, these invertebrates visit the flowers, carry and transfer mucilage mass with embedded pollen from the male flowers to the stigmas of the female flowers. Pollen tubes are formed on the stigmas, indicating that pollination is successful. Thus, *T. testudinum* has mixed abiotic–biotic pollination. We propose a zoobenthophilous pollination syndrome (pollen transfer in the benthic zone by invertebrate animals) which shares many characteristics with hydrophily, but flowers are expected to open-up during the night.

## Abstract (web summary)

Marine plants reproduce by hydrophilly, that is, the movement of pollen by water. Here, the authors show that invertebrates can also carry pollen from male to female Thalassia testudinum plants.

## Graphical abstract

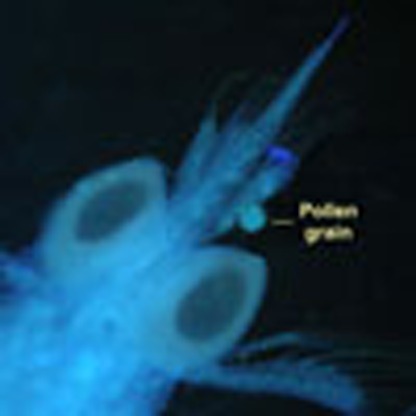

Pollination is a key process in the life cycle of flowering plants (angiosperms). It typically involves a vector responsible for the transport which can be either biotic (animals) or abiotic: usually wind. Another abiotic vector is water-flow beneath the surface of the water, which is named true hydrophily (or hydrophilic pollination). Hydrophily only occurs in 14 out of the ∼14,000 angiosperm genera, and 10 out of these 14 genera occur in the marine environment^1^.

Flowering plants in the marine environment are commonly known as seagrasses^2^. All seagrass species, but one, are truly hydrophilic^1^; although some can also pollinate at the water surface when growing in or just below the intertidal zone^3^. Seagrasses form extensive meadows in shallow marine waters; and are amongst the world's most productive ecosystems. They improve water transparency, stabilize coastlines and store carbon, and also provide food and shelter to a diverse faunal community^4^. Seagrasses are clonal plants, and flowering has long been considered of little importance in this group of plants^5^. But gradually, this paradigm of the insignificance of sexual reproduction has been changing. Newly developed genetic markers have revealed that seagrass populations can be genetically highly variable. In addition, sexual reproduction is important for re-colonization after major disturbances and maintaining the gene-flow among populations^6^. However, knowledge of the processes involved in the reproduction and pollination of seagrasses is still limited^1^, which can partly be attributed to the inconspicuousness of the flowers^2^. In addition, seagrass flowers are ephemeral once they opened-up, which may be a consequence of flowering in an aqueous medium; alternatively, it may be an adaptation to synchronize reproduction optimizing the chance of fertilization^7^ and/or avoid predation by fish^8^.

A suite of floral adaptations is associated with the water-mediated pollen transport, such as separation of the male and female structures in different flowers on the same plant (monoecy) or different plants (dioecy), and reduced perianths. The female flowers have tentacle-like stigmas, and the male flowers produce copious pollen. Pollen with a reduced exine layer, is either elongated (filiform) or spherical released in elongated strands of mucilage^1^,^3^,^5^,^7^. *Thalassia testudinum*, the species under study, is the dominant seagrass throughout the Caribbean and subtropical western Atlantic. It is dioecious with separate male and female plants with flowers situated 1–2 cm above the sea-floor within the canopy. Male flowers occur in clusters of one to five flowers (usually two or three) and produce in the order of 1.6 × 10^5^ pollen per flower^7^. Pollen grains (diameter ∼56 μm) are released in strands or masses of almost neutrally buoyant mucilage^7^,^9^. Female flowers usually occur singly with an inferior ovary below sediment level that develops into a relatively large fruit (diameter 20–25 mm) with 1–6 (usually 2–4) seeds. The male flowers open-up at sunset and release the pollen within 1–2 h at night^9^,^10^. The female flowers open-up during the day and are viable up to 72 h (ref. 7).

Until recently, it was thought that in the sea, unlike terrestrial environments, animals are not involved in pollination. However, van Tussenbroek *et al*.^11^ describe a highly abundant and diverse faunal community visiting the flowers of *T. testudinum* at night. Seawater is almost 800 times as dense as air; and small crustaceans (0.6–8 mm long; mostly <1 mm) and polychaetes (1−20 mm long; mostly <10 mm) are swept by water motion. But they show directional movements when approaching and visiting the male flowers, probably being attracted to the mucilage-pollen mass, mainly composed of nutritious polysaccharides and proteins^11^. These visitors can be a potential vector of pollen transfer between flowers; and for a visitor to be confirmed as a pollinator, the following conditions should be demonstrated^12^: (1) both male and female organs are visited, (2) the visitor carries pollen, (3) the visitor transfers pollen between male and female sexual organs, (4) pollen deposition by the visitor results in successful fertilization, estimated as pollen germination on the stigmas, pollen tube growth or seed set.

We tested these four requirements to confirm whether the visiting invertebrates were pollinators on *T. testudinum* in three different experimental set-ups. The main challenge to disclose whether the fauna potentially pollinates this seagrass is excluding pollen transfer by water. We achieved this by placing flowers and fauna in small aquaria or mesocosms without water-flow (Supplementary Figs 1 and 2). Before each trial, the fauna was captured with 1.6 l light traps after sunset. The first set-up served to observe visitation behaviour of fauna, and deposition of pollen on the stigmas. Recently dehisced male and female flowers were placed 2–3 cm apart in an aquarium, and filmed in absence or presence of abundant fauna (density≈500 individuals per liter). The aim of a second aquarium set-up was to verify attractiveness of the female flowers to fauna. Visits to female flowers were registered on video, in absence or presence of water movement (generated with two small powerheads), with a foliar shoot of *T. testudinum* as control substrate. A third set-up tested pollination success in a more natural setting in mesocosms (∼100 l) with or without fauna (density ∼30–90 individuals per liter). Male- and female flowers were placed 15 to 150 cm apart (corresponding to distances in a meadow with relatively abundant flowering) to determine if the proximity of a male flower was determinant in the success of pollination. The flowers were left in the mesocosm during the night. Afterwards, the female flowers were removed and left in a separate tank to permit the growth of pollen tubes, which were detected in preserved stigmas and styles under a fluorescent microscope after staining^13^.

With this experimental evidence we demonstrate that marine invertebrates are pollinators of *T. testudinum*. They visit both female and male flowers, carry pollen grains on their bodies, and transfer pollen between male and female flowers in aquaria experiments. In the mesocosms, the transferred pollen grains germinate on the stigmas and form pollen tubes, indicating successful pollination. Thus, *T. testudinum* has both hydrophilous and zoophilous (transport by animals) pollination, revoking the paradigm that pollen in the sea is only transported by water.

## Results

### Both male and female organs are visited

**Figure 1:**
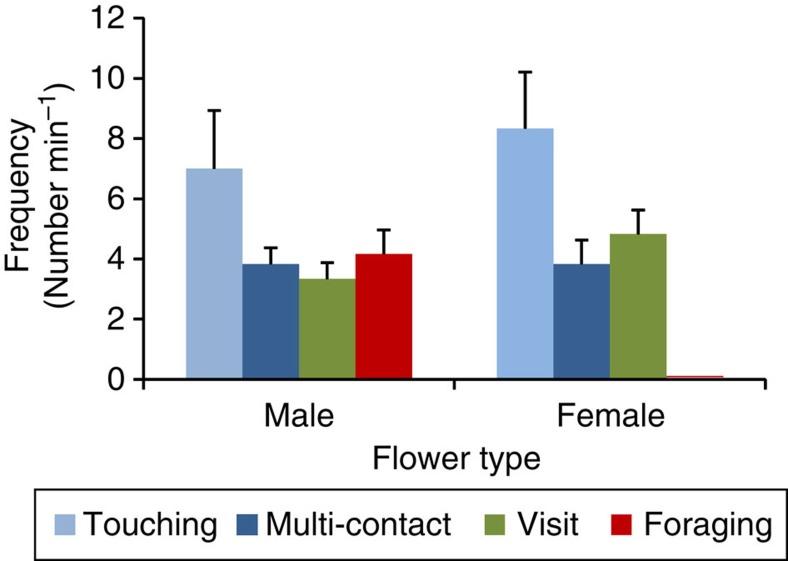
The frequency of the visits to male or female flowers of *Thalassia testudinum*. One male and one female flower were placed together in an aquarium and filmed in six trials at high faunal density (∼500 individuals per liter). The result of the Chi-squared analysis was: *χ*^2^=26.99, df=3, *P*<0.001; rejecting H_0_ (the number of visits of each type is independent of the sex of the flower). Mean (±s.e.m.), *n*=6.

**Figure 2:**
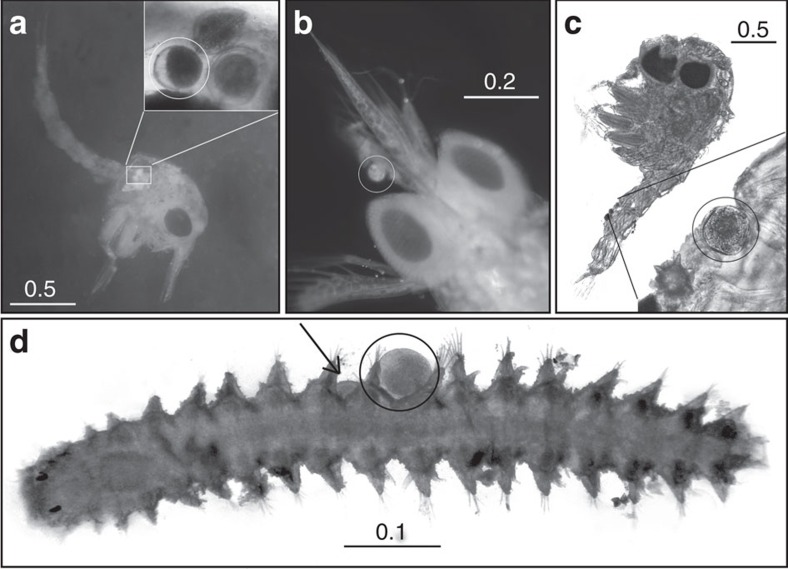
Fauna with pollen grains. (**a**) Majid zoea larva (stage I) with a pollen grain in digestive tract (detail was obtained with different illumination). (**b**) *Thallasinidea* zoea (stage I) with a pollen grain near rostrum and antenna. (**c**) Brachyuran zoea (stage I) with a pollen grain attached to abdomen. (**d**) Young Syllid polychaete with two pollen grains attached to segments; the almost hidden second grain is indicated by arrow. The pollen grains are indicated by circles. The bars represent mm.

**Figure 3:**
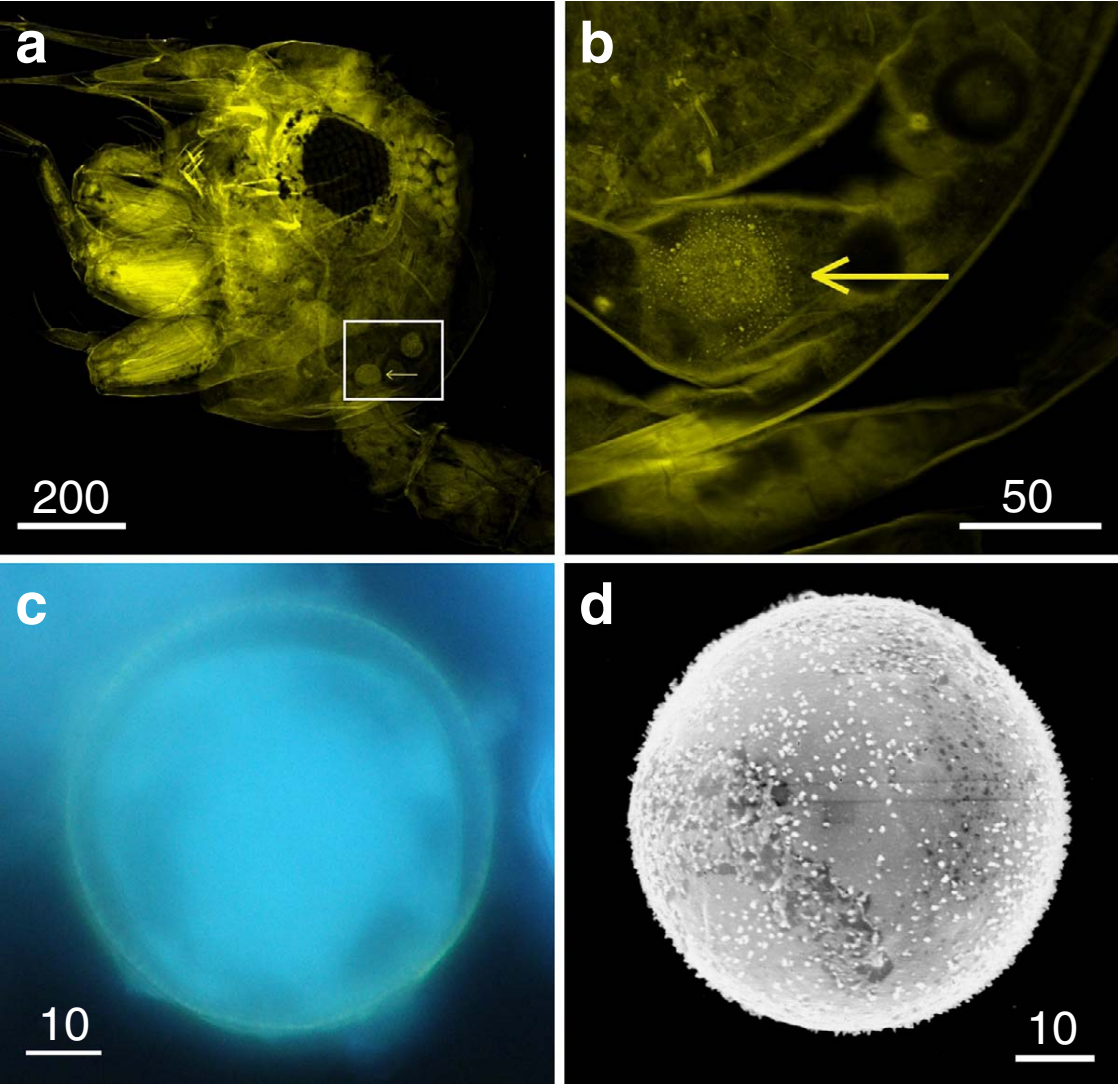
Tests for verification of pollen grains of *Thalassia testudinum*. (**a**,**b**). Pollen grain (indicated by arrow) in the digestive tract of a decapod zoea I (Brachyura), stained with auramine-O. Images were taken with confocal microscope with excitation wavelength 405 nm and emission wavelength 422 nm (the chitin of the exoskeleton of crustaceans exhibits natural fluorescence). In the detailed image (**b**), the microechinate ornamentation lights up as yellow points. (**c**) Pollen attached to the abdomen of a crustacean stained with auramine-O which lights up the exine in yellow, under fluorescent microscope. (**d**) Photograph of pollen grain with a scanning electron microscope showing the microechinate ornamentation. The bars represent μm.

Confirming contact with the reproductive organs of the flowers is the first step towards proving that a visitor is a pollinator. In the first experimental set-up, comparing the behaviour of fauna on male and female flowers in aquaria, we identified four types of visitation behaviour: (1) touching: the fauna touched the plant parts, the contact only lasting a fraction of a second; (2) multi-contact: the fauna touched these the parts at least two times consecutively; (3) visit: the fauna settled for >1 s on the parts; (4) foraging: behaviour indicating feeding; either by moving along the plant parts or exhibiting abrupt movements of retreat. The first three behaviour types were witnessed on both male and female flowers; however, foraging was only observed on male flowers (Fig. 1). We identified spheres in the digestive tract of the transparent zoea (Fig. 2a). The shape and size of several spheres corresponded with that of pollen of *T. testudinum*, which was confirmed by histochemical staining with auramine-O (Fig. 3), because exine exhibits fluorescence with this staining technique^14^. The pollen grains in the digestive tract of the crustacean larvae indicated that they ingested the mucilage-pollen matrix.

**Figure 4:**
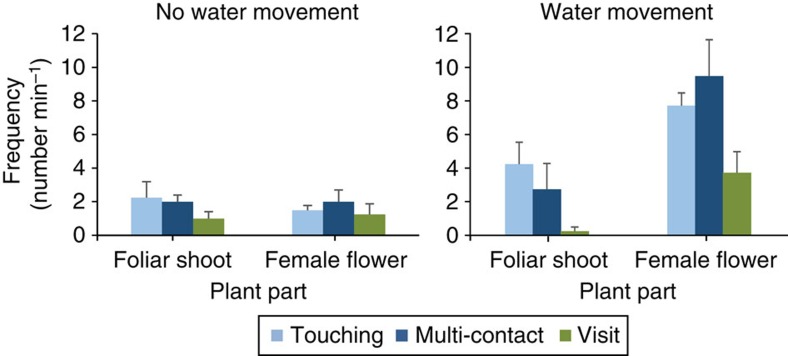
The frequency of visits to female flower or foliar shoot of *Thalassia testudinum*. The female flowers and foliar shoots in aquaria were filmed in absence and presence of water movement in four trials (Faunal density ∼100 individuals per liter). In absence of water movement, the result of the *χ*^2^ analysis was *χ*^2^=0.13, df=2, *P*=0.993; failing to reject H_0_ (The number of visits of each type is independent of plant part). With water movement, *χ*^2^=9.26, df=2, *P*=0.001; rejecting H_0_. Mean (±s.e.m.), *n*=4.

In the second aquarium experiment, we tested the attractiveness of the female flowers to fauna in comparison with vegetative plant parts. Although both flowers and foliar shoots were visited, there was no difference in the number and type of visit to the female flowers and foliar shoots in absence of water movement. However, at water movement, the female flowers were visited more frequently than the foliar shoots (Fig. 4), indicating that under natural conditions with flow, these flowers can effectively capture small fauna. The stigmas are very sticky, and *in situ* small sand grains, debris and fauna remain stuck until swept away by water movement (Supplementary Fig. 3).

### The visitor carries pollen

Fauna caught in the light traps consisted mainly of small crustaceans and polychaetes (Supplementary Table 1). Some specimens, collected after termination of the mesocosm experiments, had one or many spheres resembling pollen attached to their bodies. Crustaceans had spheres on antennae, mouthparts (maxillipeds), pereopods or abdomen (Fig. 2b,c), and polychaetes usually had many attached to their segments or bristles (chaetae, Fig. 2d). Histochemical staining with auramine-O confirmed that these spheres were pollen grains (Fig. 3c).

### The visitor transfers pollen between male and female organs

**Figure 5:**
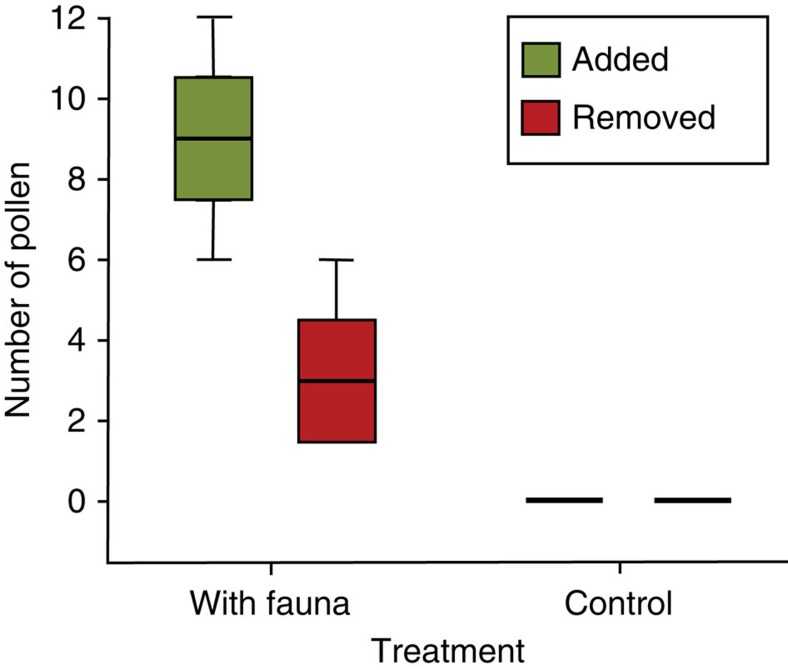
Pollen transfer by fauna. Boxplot of the number of added and removed pollen grains on the stigmas of *Thalassia testudinum* in the first aquaria experiments with (*N*=6) or without fauna (control, *N*=4), after 15 min. The horizontal bars are the median, the boxes show 25th and 75th percentiles, and the whiskers indicate spread. No pollen was added or removed in control treatments without fauna.

Deposition of pollen on the stigmas by fauna was demonstrated in the first experimental aquaria set-up observing the fauna on a male and female flower. We counted the number of pollen grains on stigmas at the beginning and after 15 min. When we initiated our observations, most female flowers had already some grains attached; most likely deposited during manipulation of the flowers. After 15 min, on average 9 (±3.9 s.e.m., *N* 6) grains were added, and 3 (±0.8 s.e.m., *N* 6) grains were removed from the stigmas of the flowers (Fig. 5). Only fauna could have moved the pollen because there was no water-flow in the aquaria. No pollen grains were gained or lost on the stigmas of female flowers in the control treatments without fauna (Fig. 5).

### The deposited pollen results in successful pollination

**Figure 6:**
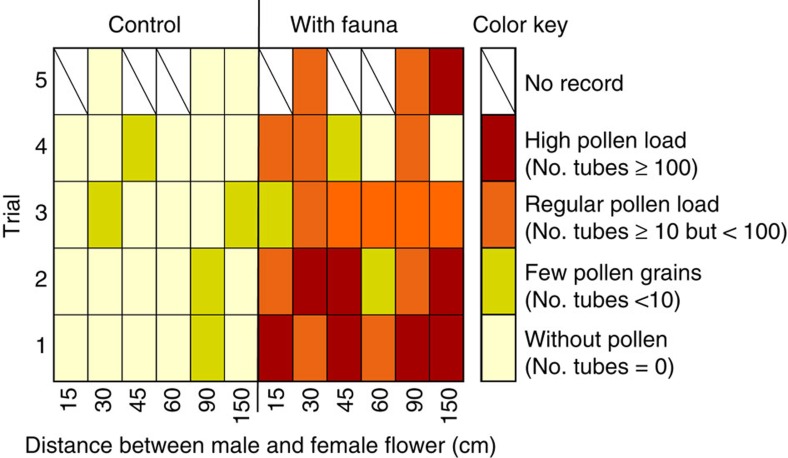
Developed pollen tubes in absence or presence of fauna. Heatmap of the abundance of pollen tubes in female flowers of *Thalassia testudinum* in the mesocosms in absence (control) or presence (with fauna) of fauna. The male and female flowers were separated by a range of distances from 15 to 150 cm.

In the third experimental set-up with the mesocosms, the pollen transported by fauna resulted in successful pollination, as most female flowers showed pollen tubes (Fig. 6; Supplementary Fig. 4). The control treatments without fauna, in contrast, had only few pollen tubes on rare occasions (Fig. 6). We expected to find no pollen tubes at all in the control treatments, because the flowers dehisced after we placed these in the mesocosms; however, they could have been from pollen grains left over in the tanks or introduced with the newly collected male shoots, since some male shoots had a flower that had opened-up the previous night with possibly some remnants of pollen, and ∼1% of pollen grains is still viable after 24 h (ref. 10). But the number of pollen tubes on the control flowers was always minimal, and it was significantly different from the number on the female flowers in presence of fauna (*χ*^2^ 38.75, df 4, *P*<0.001). In the tanks with fauna, distance between male and female flowers had no significant effect on the number of pollen tubes (*χ*^2^ 10.465, df 15, *P*>0.90; Fig. 6), suggesting that fauna can transport the pollen over relatively large distances.

## Discussion

In this study, we show that the invertebrate fauna visiting the seagrass *T. testudinum* comply with the four prerequisites to be considered pollinators. The pollination process involves the drifting fauna approaching the mucilage-pollen mass of the male flowers. The fauna forages on this mass, and some pollen grains remain attached to their body parts due to the sticky nature of mucilage. The fauna removed from the flower by water movement is then captured by the tentacle-like stigmas of the female flowers, and pollen grains are deposited to subsequently germinate, forming pollen tubes. The mucilage of the male flowers forms a cloud when dissolving in the water, increasing viscosity of the water and decreasing flow velocity^15^; thereby assisting in the approach of the fauna. The mucilage-pollen mass is a likely food reward for the pollinators, in exchange for pollen dispersal services similar to many terrestrial plants^16^. The tentacle-like stigmas and bracts of the female flowers change the water-flow patterns facilitating the capture of pollen^1^ and fauna, which subsequently remain stuck to the stigmas. Pollination also occurs in the absence of water movement in the mesocosms, suggesting that the fauna can move actively towards both male and female flowers in the absence of flow, possibly in search for substrate for settlement or driven by chemotaxis (movement driven by a chemical stimulus)^17^.

We do not discard water as the principal vector for pollen transport in this seagrass species. But similar to many wind-pollinated terrestrial plants that have shared traits with insect-pollinated plants^18^, *T. testudinum* likely has a mixed pollination syndrome: hydrophilous and ‘zoobenthophilous'. Zoobenthophily is a new type of pollination derived from benthos (community of organisms that live in, near or on the sea-bottom), because the pollination occurs near the seabed and many invertebrates belong to zoo-benthos; although zoea are free-swimming or free-floating^19^, and occur both in the benthic and planktonic zone. Many characteristics of the zoobenthophilous pollination syndrome coincide with that of hydrophily; such as pollen release in abundant mucilage and tentacle-like stigmas. However, similar to nocturnal flowering of plants associated with pollination by bats, beetles or moths^20^,^21^, we expect nocturnal pollen release to be a trait of the zoobenthophilous pollination syndrome. Many small pollinating invertebrates hide in the sediment and in the seagrass canopy during the day, and become active at night^22^,^23^. Very little is known about the timing of pollen release in seagrasses, and the only records of other seagrass species with nocturnal opening-up of male flowers (in aquaria) concern *Halophila hawaiiana*^24^.

Zoobenthophily likely enhances the reproductive success of the seagrass *T. testudinum*. It can be a mechanism of reproductive assurance in absence of water movement (although this is uncommon in the marine environment), and it likely extends the water-mediated pollination range which is limited^25^. At distances >20 cm between male and female flowers, the probability of fertilization already decreases^26^. Marine invertebrates with semi-active locomotion^27^ can travel much further, potentially extending the dispersal range of pollen. This is confirmed by our results from the mesocosm experiment with fauna, where the distance between male and female flowers (range 15–150 cm) had no effect on the number of developed pollen tubes. In this context, it is important to mention that our light traps only captured the smaller invertebrates, as the larger ones usually escaped from the traps. We registered a higher frequency of larger specimens in the field^11^, and we observed that larger specimens carried more pollen (www.youtube.com/watch?v=B7VLBhQ-rQo); thus, we expect that the contribution of fauna to pollen transport in our experiments was underestimated. Negative consequences of the consumption of pollen grains by the invertebrate fauna are thought to be negligible, as only a very small fraction of the 1.6 × 10^5^ pollen grains per flower is consumed. The actual contribution of zoobenthophily versus hydrophily in the pollination success of this seagrass has yet to be established in the sea, and will most likely depend on environmental settings.

Pollination by water-flow is uncommon, and most freshwater aquatic plants have flowers that emerge into the air^28^. Marine angiosperms probably evolved from freshwater ancestors^1^,^3^,^29^, and the Hydrocharitaceae (the family of *T. testudinum*) were likely insect pollinated^28^. Terrestrial abiotic pollination usually occurs by wind, which probably evolved from insect pollination in response to pollinator limitations and changes in the abiotic environment^18^. Similar to insect pollination, pollination by invertebrate marine fauna may have played a role in the transition from terrestrial to abiotic submarine pollination by water-flow. However, seagrasses are polyphyletic^30^ and the transition from freshwater to the marine environment may have differed among lineages. Further investigations into the reproductive ecology of this group of plants may reveal whether mixed abiotic–biotic pollination syndrome occurs more frequently in the marine environment.

## Methods

### Collection of flowers

The experiments were performed from 20 to 24 April 2014, and 13 to 14 May 2016. At midday, before the experiments*, T. testudinum* foliar shoots with closed male or female flower buds were collected at different sites with abundant flowering in Puerto Morelos reef lagoon, Mexico. Only buds extending above the sediment level having a stretched pedicel were selected, because they were expected to open-up that same evening^10^. Foliar shoots with the buds for the aquarium experiments, were cut with a knife below substratum, and they were kept separately in closed seawater tanks until used. Flowers for the mesocosm experiments were sampled with a PVC corer (diameter 4.3 cm, 15 cm depth), to collect a whole flowering shoot with a small sod of sediment.

### Collection of fauna

Invertebrate fauna was collected immediately before each experiment. Collection occurred after sunset (after 20:30 local DLS time), in home-made traps of 1.6 l of transparent plastic flasks, with inverted entrance, tied to a rod with a diving lamp. The light of the diving lamp attracted the fauna, and the traps were left 30–40 min above a near shore seagrass meadow. This meadow had none or very few flowers to avoid collecting fauna with pollen attached as much as possible.

### Aquarium experiment 1

*T. testudinum* flowers and fauna were observed in small aquaria placed in the dark. The seawater with fauna from a trap was very carefully poured into the aquaria (Supplementary Fig. 1) and filled with additional seawater until 3 l. The density of organisms in the aquaria was ≈500 individuals per liter; the majority being small crustacean larvae (Supplementary Table 1). The flowers were presented in pairs: the first flower always was a recently opened male flower with abundant pollen embedded in mucilage, and the second flower a recently opened female flower. The flowers were placed in small trays (5 × 6 cm), divided in two sections with a 3 cm high separation, to avoid pollen transport between the flowers during manipulation when placing the flowers (especially the sticky mucilage of the male flowers is difficult to handle). The trays were introduced into the aquaria with fauna (Supplementary Fig. 1). We conducted six trials with different flowers and fauna. Both flowers received equal illumination to allow filming during 15 min. But only the first minute of each film was analysed for behaviour, because some organisms were trapped in the sticky mucilage mass of the male flowers in the absence of water movement. We determined the number of visits per flower, and they were added for all female or male trials, and a *χ*^2^ analysis was carried out to test whether the type of visits was independent of the flower type (female versus male).

The number of pollen on the female flowers was counted at the beginning of the experiment and after 15 min. We also included four control treatments without fauna.

### Aquarium experiment 2

The aquaria were prepared as above, with one female flower and one foliar shoot of *T. testudinum*, and two small powerheads to induce water movement (Supplementary Fig. 1C). Either the female flower or the foliar shoot was placed in the centre and filmed during 1 min; with and without current (powerheads on or off). This was repeated four times with different flowers, shoots and fauna. The types of visits were registered as above.

### Experimental trial 3 with mesocosms

Experiments were carried out from 7 until 24 of April 2013 in Y-maze mesocosms (1.7 × 0.8 × 0.4 m depth, length partial separation 1.0 m, Supplementary Fig. 2). The mesocosms were filled with seawater from a closed water circuit treated with UV light, to eliminate the possible influx of external viable pollen. The cores with flowers were transported within 1–1.5 h of collection to the mesocosms and the sods with the shoots were placed in concrete blocks of 15 × 15 × 17(h) cm with a central hole of the size of the core samples. Four shoots (two male and two female) were placed per mesocosm, and there were four mesocosms in total. Similar treatments were applied to all flowers in the same mesocosm. The treatments (distance, with fauna or control), were assigned randomly to the mesocosms. The distances between the male and female flowers were 15, 30, 45, 60, 90 and 150 cm on different nights. The female flowers were always placed at the end of the separation of the Y-maze and the position of the male flowers was changed depending on the treatment. The female flowers in the same mesocosm were treated as independent replicates, which was reasonable because the flowers in the same tanks received different quantities of pollen. Seawater with fauna from two 1.6 l traps, was very carefully poured into the mesocosm (of the fauna treatment) away from the male flowers. The density of the fauna in the mesocosms varied between ≈30 and 90 individuals per liter. For each distance there was a control (no fauna) and a faunal trial. The control trials received the same volume of seawater. The flowers were left overnight (from ∼22:00 until 05:30–06:00 next morning) in the dark. The following morning, the concrete blocks with the shoots bearing the female flowers were carefully removed from the mesocosms and placed in separate tanks (0.6 × 0.6 × 0.3 m depth) for at least 16 h to allow for the growth of the pollen tubes before they were fixed in FAA (formalin–acetic–alcohol). The fauna was collected in sieves when water was removed from the tanks, and ∼5% was analysed to determine the composition of the fauna until Family level, and to check whether they had pollen grains attached.

The treatments were rotated among the mesocosms randomly after thorough cleaning. The floral buds did not always open-up; therefore, the number of replicates might vary per treatment.

### Observation on pollen tubes

The pollen tubes, in squash preparations of stigmas and styles of the fixed flowers, were detected under a fluorescent microscope after staining with anile-blue^13^ (Supplementary Fig. 4). It is expected that if the fauna transports pollen, pollen tubes will be registered in the female flowers, as hydrophilous pollination is unlikely due to the absence of water movement.

When pollen tubes are abundant it is difficult to determine their exact number in the stigmas and style of the female flower, therefore we established the following categories: 0: without pollen tubes, 1: less than 10 pollen tubes, 2: regular amount of pollen tubes (>10 but<100), 3: many pollen tubes (>100). *χ*^2^ Analysis was carried out to test if the abundance of pollen tubes in the female flowers was independent of treatment: pooled data for all mesocosms with or without fauna (Control). Another *χ*^2^ analysis was carried out to determine whether the abundance of pollen tubes in the female flowers was independent of the distance between the male and female flowers for the mesocosms with fauna.

### Invertebrate fauna with pollen

The fauna from the mesocosms was sampled after conclusion of the experiments, and fixed in alcohol. The principal groups were identified (Supplementary Table 1), and specimens with attached spheres resembling pollen were separated. Fauna from the aquaria was fixed in alcohol within 10–15 min after termination of the experiments, and the gut contents of the preserved almost transparent zoeas were examined. We applied the stain auranina-O, a fluorescent dye that only stains exine and lights up under fluorescent light, to verify whether the spheres inside or attached to the fauna were pollen grains. Spheres inside a zoea suspected to be pollen were examined under a confocal microscope (Olympus FV 1000) with excitation wavelength 405 nm and emission wavelength 422 nm. Selected fauna with spheres attached were observed under a fluorescent microscope (Olympus BX41).

### Data availability

The data that support the findings of this study are included within the Article and Supplementary Information Files or available from the authors upon request.

## Additional information

**How to cite this article:** van Tussenbroek, B. I. *et al*. Experimental evidence of pollination in marine flowers by invertebrate fauna. *Nat. Commun.* **7, **12980 doi: 10.1038/ncomms12980 (2016).

## Supplementary Material

Supplementary Information Supplementary Figures 1-4 and Supplementary Table 1
