# Supplementary material for: Experimental evidence of pollination in marine flowers by invertebrate fauna
Source: Nat Commun. 2016 Sep 29;7:12980. doi: 10.1038/ncomms12980 (PMC5056424; doi:10.1038/ncomms12980)
Supplement: Supplementary Information — Supplementary Figures 1-4 and Supplementary Table 1 [file ncomms12980-s1.pdf]

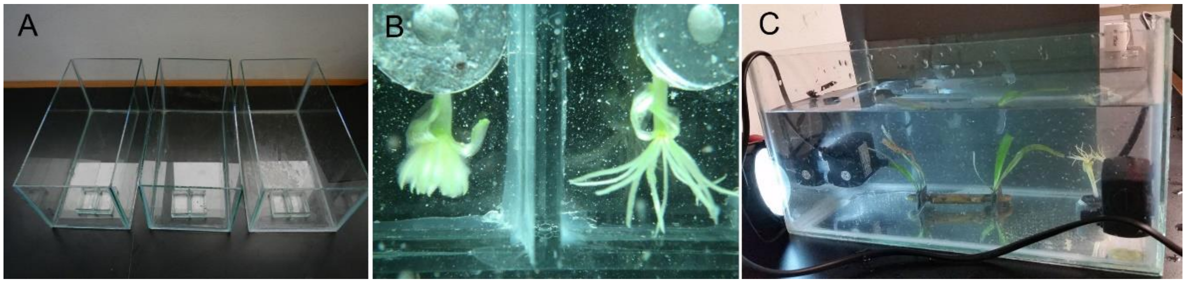

Supplementary Figure 1: A. Aquaria with trays for *Thalassia testudinum* flowers, B Tray with pollen-producing male (left) and female flower (right), C. Aquarium with female flowers, foliar shoots and powerheads (flowers and shoots not in the position of the experiments)

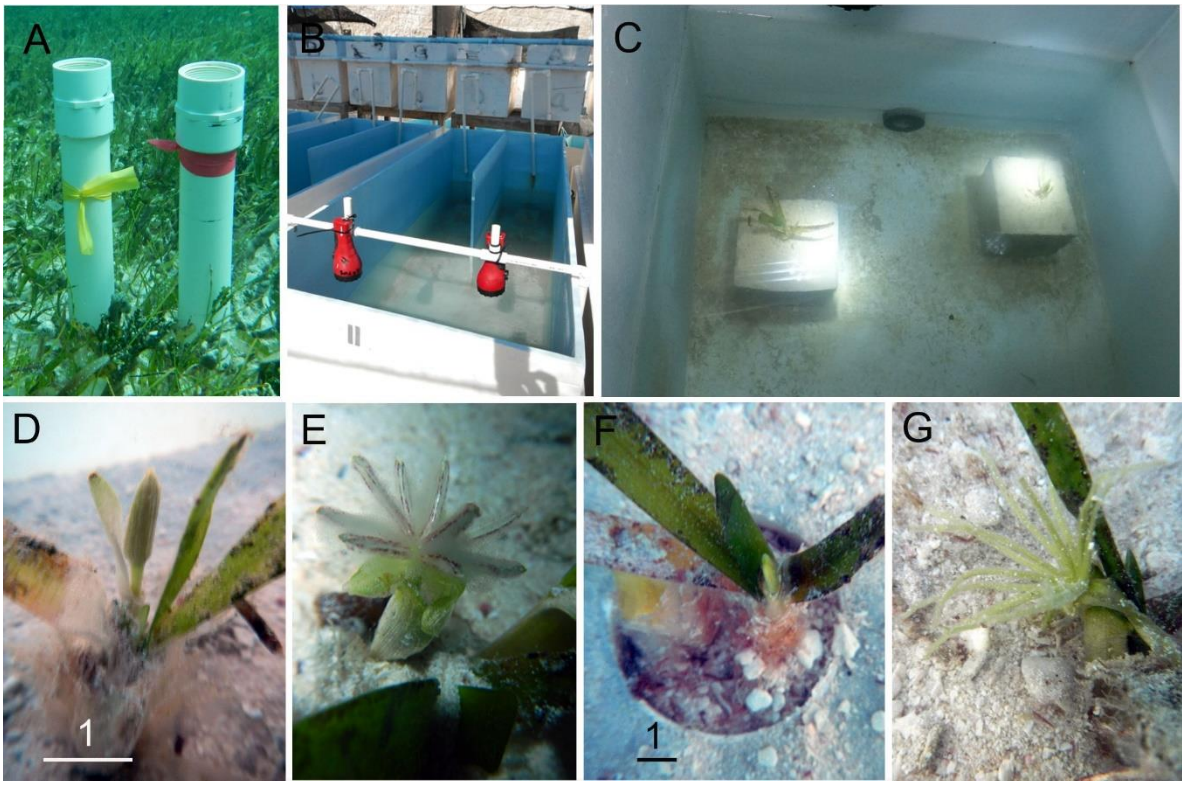

Supplementary Figure 2: The mesocosm experiment: A. Sampling of shoots with floral buds during the day, B. Preparation of mesocosms: the female flowers were placed at the extreme end of the Y. The lamps were only used for verification whether the flowers had opened-up at the beginning of the experiments, C. Blocks with male flowers opening-up during the night, D. Male flower bud, E. Male flower releasing pollen, F. Female flower bud, F. Female flower recently opened-up. Scale in cm.

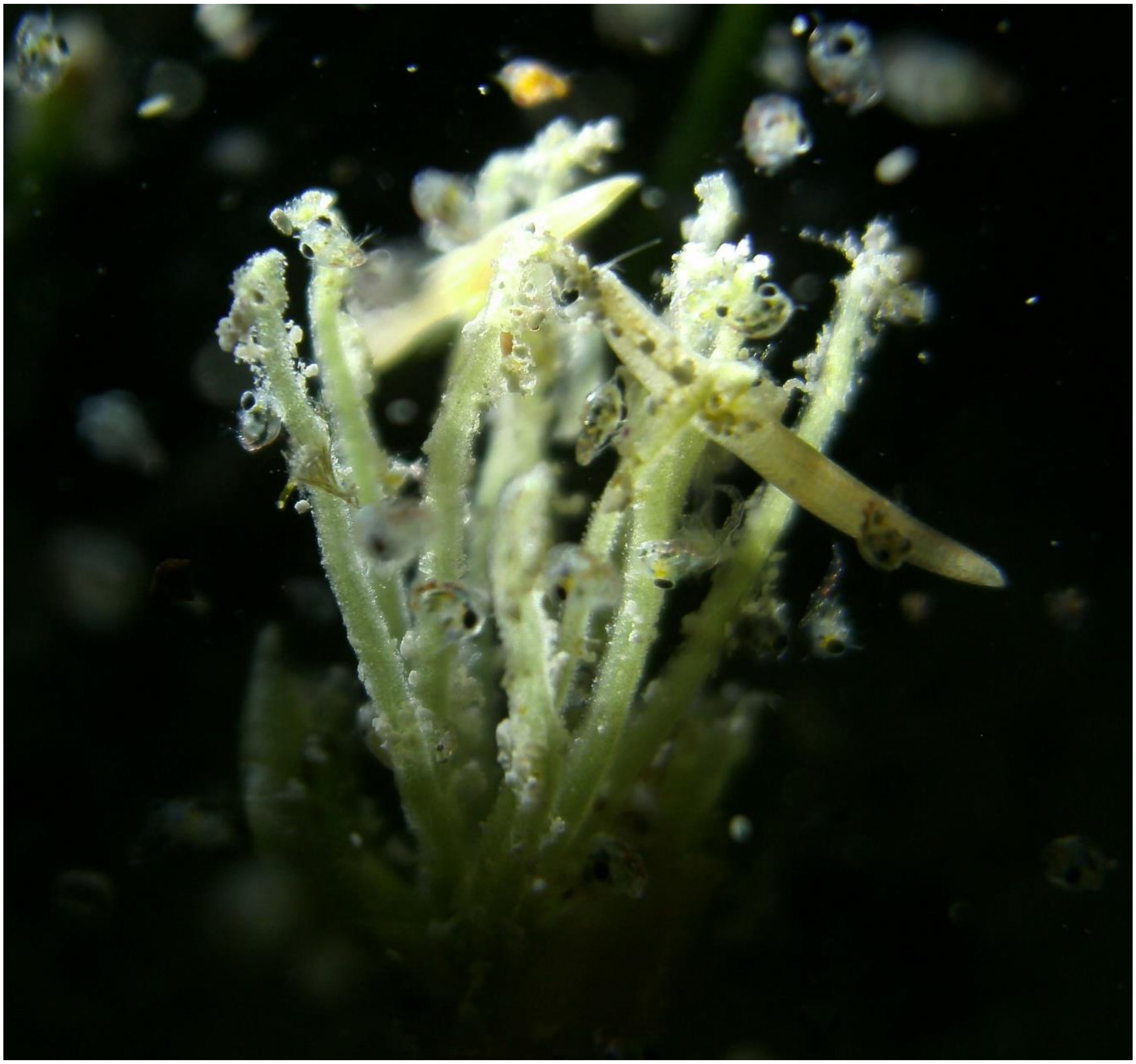

Supplementary Figure 3. *In situ* image of a female flower of *Thalassia testudinum* with visiting fauna (polychaetes and crustacean larvae), showing sticky stigmas with attached sand grains. The density of the fauna was higher than usual, because they were attracted by the light required for filming.

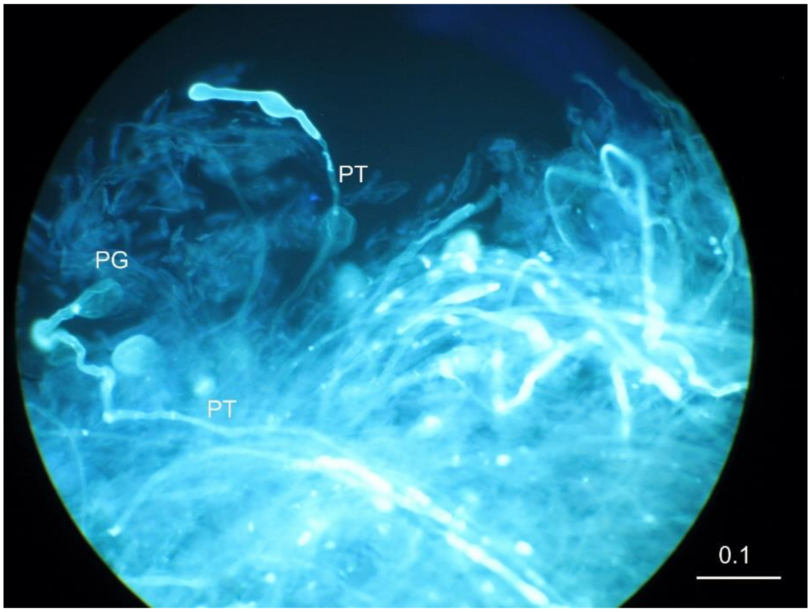

Supplementary Figure 4. Squash preparation of stigmas and style of *Thalassia testudinum* under a fluorescent microscope. Germinated pollen grains with pollen tubes were stained with aniline-blue which evidences the callose in the pollen tubes[14]. PG (empty) pollen grain, PT pollen tube. Scale in mm.

Supplementary Table 1. List of principal faunal groups sampled in the light traps. Abundance was estimated from subsamples. Visitors in the field to male and female flowers from Van Tussenbroek et al. (2012). Juv. juvenile

Faunal group	Estimated Abundance	Observation (*most abundant species within the taxon*)	Visitor to male or female flowers *in situ*	Average Size (mm)
**Phylum Chaetognatha**				
-*Sagitta sp.*	< 1%		No	12
**Phylum Scyphozoa**				
-various jellyfishes	< 1%		No	8
**Class Polychaeta**				
- Nereididae	<1%	(*Rullierinereis mexicana*)	Yes	14
- Alciopidae	<1%	(*Rhynchonerella petersi)*	Yes	10
- Syllidae	3%	*(Salvatoria sp.*, juv.)	Yes	3
**Subphylum Crustacea**				
Class Maxillopoda				
-*calanoid copepods*	12%	(*Acarthia sp*.)	Yes	1.5
Class Ostracoda				
Cypridinidae	< 1%	(*Skogsbergia sp*. & *Kornickeria sp*.)	Yes	3
Class Malacostraca				
Superorder Peracarida				

-Mysidacea	< 1%		Yes	6
-Amphipoda	< 1%	(*Tethygeneia sp.*)	Yes	5
-Isopoda				
Bopyridae:	10%		Yes	1
-Tanaidacea				
Leptochellidae	< 1%	(*Leptochelia dubia*, juv.)	Yes	3
-Cumacea				
Nannastacidae:	< 1%	(*Cumella sp.*)	Yes	1.7
Order Stomatopoda				
stomatopod shrimps	7%	Larvae	No	13
Order Decapoda	65%	Zoea larvae		
Infraorder Caridea	-	Zoea I	Yes	4
Infraorder Thalassinidea	-	Zoea I	Yes	5
Infraorder Brachyura	-			
-Majidae		Zoea I	Yes	8
